# Knowledge, attitude and perceptions of pharmacists regarding renal dose adjustment among chronic kidney disease patients in Pakistan

**DOI:** 10.1186/s40545-023-00606-4

**Published:** 2023-09-19

**Authors:** Roheena Zafar, Inayat Ur Rehman, Yasar Shah, Zahid Ali, Long Chiau Ming, Tahir Mehmood Khan

**Affiliations:** 1 Department of Pharmacy, Abdul Wali Khan University Mardan, Mardan, 23200 Pakistan; 2 Department of Pharmacy, Northwest General Hospital and Research Centre, Peshawar, 25100 Pakistan; 3 Department of Pharmacy, University of Peshawar, Peshawar, 25120 Pakistan; 4 School of Medical and Life Sciences, Sunway University, 47500 Bandar Sunway, Malaysia; 5 Institute of Pharmaceutical Sciences, University of Veterinary and Animal Sciences, Lahore, 54000 Pakistan

**Keywords:** Knowledge, Attitude, Perception, Renal dose adjustment, RDQ-13, Pakistan

## Abstract

**Background:**

Chronic kidney disease (CKD) poses a significant public health challenge. CKD patients have compromised renal function, which not only alters the pharmacokinetics of drugs but also their pharmacodynamics. Adjusting drug doses for these patients is essential to achieve the intended clinical outcomes, prevent adverse drug events, and halt further progression of the disease. Pharmacists play a pivotal role in ensuring safe and appropriate therapy for CKD patients. However, there is a noticeable absence of national dosing guidelines for CKD in Pakistan, coupled with a scarcity of studies exploring the knowledge, attitude, and perception of renal dose adjustments in the country. This study aimed to evaluate the knowledge, attitudes, and perceptions of pharmacists in the Khyber Pakhtunkhwa province and Islamabad regarding renal dose adjustments.

**Methodology:**

A cross-sectional study was conducted to gauge the knowledge, attitude, and perception of pharmacists working in various cities of Khyber Pakhtunkhwa and the capital city, Islamabad, from February to May 2023. The Renal Dosing Questionnaire-13 (RDQ-13) scale was employed for this purpose. The survey link was disseminated through emails, and the RDQ-13 scale was also completed in person by pharmacists from hospitals, clinics, community, and retail settings who interact with CKD patients. Univariate linear regression was employed, and factors with a *p* value < 0.25 were subjected to multivariate linear regression. For comparing knowledge, attitude, and perception scores of pharmacists, the independent *t* test and one-way ANOVA were utilized as appropriate. A *p* value < 0.05 was deemed statistically significant.

**Results:**

Of the 384 pharmacists approached, 270 completed the RDQ-13 scale, resulting in a response rate of 70.3%. The overall knowledge score regarding renal dose adjustment was 21.24 ± 2.18 (mean ± SD). Attitude scores averaged at 10.04 ± 1.81, and perception scores at 7.19 ± 2.15. Multivariate analysis indicated a positive correlation between the pharmacists' perception scores and gender, with male pharmacists scoring higher than their female counterparts.

**Conclusions:**

The study underscores the importance of instituting targeted training programs for pharmacists, ensuring access to dependable resources, and promoting research and results dissemination in the realm of renal pharmacotherapy to enhance public health outcomes.

## Background

Chronic kidney disease (CKD) is emerging as a significant public health issue in Pakistan, with an estimated prevalence affecting 12.5–31.2% of the population [1]. A 2018 systematic review reported a 23.3% CKD prevalence in the country [2]. The disease is notably prevalent among the elderly, women, and those with comorbidities, especially hypertension and diabetes. This prevalence often results in polypharmacy, subsequently raising the potential for drug-related complications [3]. While hypertension and diabetes mellitus are established as primary drivers of CKD [4, 5], a recent Pakistani study noted associations between diabetes and hypertensive nephropathy in 27.1% and 15.2% of patients, respectively [6]. Moreover, approximately 43.6% of individuals over 50 years in Pakistan are diagnosed with CKD [7], though literature presents varied findings regarding the gender most affected by CKD [2].

In CKD, diminished renal function impacts both the pharmacokinetics and pharmacodynamics of various drugs [8, 9]. Consequently, dose adjustments are essential to attain the desired clinical outcomes, mitigate adverse drug events, and prevent disease progression [10]. Yet, even with available dosing adjustment guidelines, 25–77% of CKD patients experience inappropriate dose adjustments [11, 12]. Specifically, in Pakistan, a 2023 study found that 56.1% of medications requiring dose modifications were not aptly adjusted for CKD patients [13].

Pharmacists, integral to multidisciplinary healthcare teams, are pivotal in addressing drug-related concerns, given their clinical training. They excel in ensuring patient safety through activities, such as screening, dispensing, inspecting, counseling, and offering inpatient pharmaceutical services [14]. Multiple studies highlight the positive influence of pharmacists in managing CKD and end-stage renal disease, thereby enhancing outcomes and refining patient care [14–16].

A Japanese study observed that a lower proportion of community pharmacists (54.2%) implemented renal dosage adjustments in their daily routines compared to their hospital counterparts (91.5%) [17]. Yet, another multicenter study suggested that community pharmacists, when granted access to clinical data, appropriate training, and support from hospital-based peers with specialized knowledge, can elevate the quality of patient care [18].

Given the pivotal role pharmacists play in ensuring the safety and appropriateness of therapy for CKD patients, and considering the absence of national dosing guidelines for CKD in Pakistan, coupled with limited studies assessing knowledge, attitudes, and perceptions about renal dose adjustments, it is imperative to evaluate these attributes among pharmacists in various healthcare settings in the Khyber Pakhtunkhwa province and capital city of Pakistan, i.e., Islamabad, Pakistan.

## Methodology

### Study design, population, and setting

We conducted an observational, cross-sectional study from 1st February 2023 to 30th May 2023, aiming to evaluate the knowledge, attitude, and perceptions of pharmacists across various cities in Khyber Pakhtunkhwa and the capital city, Islamabad, Pakistan.

The inclusion criteria for the study encompassed pharmacists with a minimum education of Bachelor of Pharmacy (B. Pharm), employment in hospital, clinical, community, or retail pharmacy settings, a minimum of 1 year professional experience, and interactions with CKD patients. Pharmacists not aligning with these criteria were excluded.

### Definitions/terms used

A Knowledge, Attitude, and Practice survey is meticulously crafted to comprehensively assess a target demographic, focusing on extracting information concerning their knowledge, beliefs, and practices [19].

For our data collection, we distinguished between four primary settings: hospital pharmacy, clinical pharmacy, community pharmacy, and retail pharmacy. Their definitions are as follows:

Hospital pharmacy: A central component within healthcare facilities tasked with the procurement, conservation, formulation, distribution, manufacturing, assessment, packaging, and dissemination of pharmaceutical products [20].

Clinical pharmacy: The realm of pharmacy emphasizing the scientific basis and practical application of rational medication use and its management [21].

Community pharmacy: A healthcare entity that delivers pharmaceutical and consultative services to a designated community [22].

Retail pharmacy: Engages in supplying medications to patients and offers guidance on their proper use [23].

### Study tool

The Renal Dosing Questionnaire-13 (RDQ-13) scale is a pioneering instrument, meticulously designed to gauge pharmacists' knowledge, attitude, and perceptions concerning renal dose adjustments. Its development drew inspiration from existing literature, incorporating questions tailored to assess these three facets in relation to renal dosage modification.

The RDQ-13 integrates demographic queries (gender, age, tenure, educational qualifications, workplace setting, role, and accessible drug references). The knowledge segment spans six domains, each containing four questions answered with a binary "Yes" or "No." A "Yes" warrants a score of one, while a "No" garners a zero. The cumulative highest score across these domains is 24. Attitude appraisal employs a four-question Likert scale, resulting in a score between 0 and 3, culminating in a maximum score of 12. Perception evaluation utilizes another Likert scale with three queries: two range from 1 to 4, and one varies between 0 and 3, setting the section's ceiling score at 11. Furthermore, two supplementary questions were embedded to discern primary challenges in renal dose modification and gauge interest in pertinent courses or continuing medical education.

In terms of the RDQ-13 scale's validity and reliability, it demonstrated strong internal consistency, with a Cronbach’s alpha of 0.700. The intra-class correlation coefficient (ICC) for both initial and subsequent tests revealed significant scores for most domains (*p* < 0.001), indicating superb congruence. The Kaiser–Meyer–Olkin (KMO) value was 0.60, the Chi-square value stood at 63.430, and Bartlett’s test of sphericity was significant (df = 28, *p* < 0.001).

### Procedure

Pharmacists' consent for participation was secured after elucidating the study's objectives. For direct completion of the RDQ-13, pharmacists were personally engaged. In online scenarios, the RDQ-13 scale link was disseminated via email to pharmacists functioning in hospital, clinical, community, and retail pharmacy environments, specifically those interacting with CKD patients.

### Sample size and ethical approval

Utilizing a sample size formula [24], a requisite minimum sample of 384 pharmacists was derived, grounded on a 95% confidence interval and 5% precision rate. Ethical endorsement for this investigation was granted by the Ethical Committee of Abdul Wali Khan University Mardan, Pakistan, referenced as EC/AWKUM/2021/27/175, dated 20/11/2021. All study procedures adhered rigorously to the principles outlined in the 1975 Helsinki Declaration.

### Statistical analysis

Analyses were conducted employing SPSS version 22.0®. Descriptive statistics were deployed for demographic insights, illustrating them through frequencies and percentages. Continuous variables, such as knowledge, attitude, and perception scores of the pharmacists, were communicated via means and standard deviations.

To pinpoint determinants influencing the knowledge, attitude, and perception scores, a multivariate linear regression analysis was initiated. Scores were treated as dependent variables, with gender, age, professional tenure, educational background, operational environment, and professional title considered independent variables. Following a univariate linear regression, variables yielding a *p* value < 0.25 advanced to multivariate linear regression. The comparison of knowledge, attitude, and perception scores relative to gender, age, professional experience, education, work setting, and role leveraged independent *t* tests and one-way ANOVA where fitting. A *p* value < 0.05 was deemed to represent statistical significance.

## Results

A total of *n* = 384 pharmacists were approached out of whom only *n* = 270 pharmacists filled the RDQ-13 scale (response rate = 70.3%). Among the participated pharmacist, majority (66.7%) were males, (63.0%) were of age group of 20–30 years and (66.3%) were having professional experiences of less than 5 years. Regarding the education status of pharmacists, majority (70.7%) were having Pharm D level of education and (71.9%) were working in hospital pharmacy setting. About the designation of pharmacist working on (58.5%) were working on staff pharmacist designation and (36.7%) reported having other including Medscape, renal dosing handbook, mobile applications etc. as a drug reference available with them (details are shown in Table 1).

**Table 1:** Demographic characteristics of pharmacists included in study (*n* = 270)

	*N*	%
Gender		
Male	180	66.7
Female	90	33.3
Age in years		
20–30 years	170	63.0
31–40 years	87	32.2
41–50 years	12	4.4
51–60 years	1	.4
Professional experience		
Less than 5 years	179	66.3
5–10 years	66	24.4
11–15 years	14	5.2
More than 16 years	11	4.1
Education		
B. Pharm	5	1.9
Pharm D	191	70.7
Higher degree	70	25.9
Other professional certificate/BCPS	4	1.5
Working setting		
Hospital Pharmacy	194	71.9
Clinical Pharmacy	38	14.1
Community Pharmacy	16	5.9
Retail Pharmacy	22	8.1
Designation		
Trainee Pharmacist	31	11.5
Resident Pharmacist	25	9.3
Staff Pharmacist	158	58.5
Assistant Manager Pharmacy	16	5.9
Manager Pharmacy	25	9.3
Chief Pharmacist	10	3.7
Director Pharmacy	5	1.9
Drug reference available		
British National Formulary	48	17.8
British/US pharmacopeia	34	12.6
Lexicomp	51	18.9
Micromedex	38	14.1
Others (Medscape, Renal Dosing Handbook, mobile applications, etc.)	99	36.7

### Knowledge regarding renal dose adjustment

Based on the RDQ-13 scale's six domains, the pharmacists' responses are displayed in Table 2. The scores for each domain had a mean ± SD of 3.11 ± 0.88, 3.83 ± 0.45, 3.32 ± 0.84, 3.65 ± 0.61, 3.56 ± 0.79, and 3.77 ± 0.53, respectively. The cumulative knowledge score concerning renal dose adjustment was 21.24 ± 2.18. Considering that the maximum score was 24, this indicates that the pharmacists possessed satisfactory knowledge in this area.

**Table 2:** Responses of pharmacists on knowledge-related questions of RDQ-13 scale

	Yes	%	No	%
Domain 1: Related to renal dose adjustment				
a. Are you knowledgeable about renal dose adjustment?	241	89.3	29	10.7
b. Have you ever heard about the National Kidney Foundation KDOQI guidelines?	129	47.8	141	52.2
c. Are the medications excreted from the body through the kidneys?	267	98.9	3	1.1
d. Are you monitoring a patient's renal function during medication administration?	202	74.8	68	25.2
Domain 1: Overall score (mean ± SD)	3.11 ± 0.88			
Domain 2: Factors to be considered when determining the appropriate dose for a patient with renal impairment				
Age of the patient	258	95.6	12	4.4
Weight of the patient	258	95.6	12	4.4
The severity of the patient's renal impairment	263	97.4	7	2.6
The medication's pharmacokinetics and pharmacodynamics	256	94.8	14	5.2
Domain 2: Overall score (mean ± SD)	3.83 ± 0.45			
Domain 3: Medications commonly require renal dose adjustment				
Antibiotics	252	93.3	18	6.7
Antihypertensive	213	78.9	57	21.1
Analgesics	195	72.2	75	27.8
Other medications	236	87.4	34	12.6
Domain 3: Overall score (mean ± SD)	3.32 ± 0.84			
Domain 4: Calculating the appropriate dose for a patient with renal impairment				
Use a formula based on the patient's creatinine clearance or estimated glomerular filtration rate (eGFR)	261	96.7	9	3.3
Follow dosing guidelines provided by the medication manufacturer	225	83.3	45	16.7
Consult with a specialist, such nephrologist	239	88.5	31	11.5
Consult with a specialist, such as a pharmacist	261	96.7	9	3.3
Domain 4: Overall score (mean ± SD)	3.65 ± 0.61			
Domain 5: Consequences of not adjusting the dose of medications for patients with renal impairment				
Increased risk of adverse drug reactions	259	95.9	11	4.1
Decreased medication efficacy	202	74.8	68	25.2
Reduced quality of life for the patient	256	94.8	14	5.2
Exaggerate symptoms of disease	243	90.0	27	10.0
Domain 5: Overall score (mean ± SD)	3.56 ± 0.79			
Domain 6: Resources to determine the appropriate dose for a patient with renal impairment				
Medication dosing guidelines	261	96.7	9	3.3
Clinical practice guidelines	259	95.9	11	4.1
Pharmacokinetic information	261	96.7	9	3.3
Pharmacodynamics information	237	87.8	33	12.2
Domain 6: Overall score (mean ± SD)	3.77 ± 0.53			
Overall Knowledge score (mean ± SD)	21.24 ± 2.18			

### Attitude toward renal dose adjustment

Table 3 illustrates the pharmacists' attitude, where 96.3% considered dose adjustment for patients as very important. 44.8% felt very confident in determining the appropriate dose for patients, while 70% were very willing to seek advice from a specialist regarding medication dose adjustments. 49.3% were very open to feedback on their prescribing practices. The collective attitude score of the pharmacists was 10.04 ± 1.81.

**Table 3:** Responses of pharmacists on attitude-related questions of RDQ-13 scale

	*N*	%
How important is dose adjustment of medications for patients		
Not important	3	1.1
Somewhat important	0	0
Moderately important	7	2.6
Very important	260	96.3
How confident are you in calculating the appropriate dose for a patient		
Not at all confident	8	3.0
Somewhat confident	31	11.5
Moderately confident	110	40.7
Very confident	121	44.8
Are you willing to consult the specialist regarding medication dose adjustment		
Not willing	6	2.2
Somewhat willing	32	11.9
Moderately willing	43	15.9
Very willing	189	70.0
How receptive are you to feedback regarding your prescribing practices for patients		
Not receptive at all	7	2.6
Somewhat receptive	40	14.8
Moderately receptive	90	33.3
Very receptive	133	49.3
Overall score of Attitude (mean ± SD)	10.04 ± 1.81	

### Perception on renal dose adjustment

The pharmacists' perception scores are presented in Table 4. For the query on encountering patients with renal impairment, 31.1% responded with "frequently". 42.2% frequently adjust medication doses for such patients. Meanwhile, 52.6% felt that while there's adequate medication management for patients with renal impairment, there's still room for improvement. The combined perception score was 7.19 ± 2.15.

**Table 4:** Responses of pharmacists toward perception-related questions of RDQ-13 scale

	*N*	%
Encounter patients with renal impairment in your practice		
Rarely	38	14.1
Occasionally	70	25.9
Frequently	84	31.1
Very frequently	78	28.9
Frequently adjust medication doses for patients with renal impairment		
Rarely	33	12.2
Occasionally	63	23.3
Frequently	114	42.2
Very frequently	60	22.2
Believe that patients with renal impairment receive adequate medication management		
I'm not sure	17	6.3
Yes, medication management is adequate	79	29.3
Somewhat, but there is room for improvement	142	52.6
No, medication management could be improved	32	11.9
Overall perception score (mean ± SD)	7.19 ± 2.15	

### Comparative analysis

Table 5 highlights the comparisons:

**Table 5:** Comparison of knowledge score, attitude score and perception score against gender, age, professional experience, education, working setting and designation

	Knowledge score	*p* value	Attitude score	*p* value	Perception score	*p* value
	Mean ± SD		Mean ± SD		Mean ± SD	
Gender						
Male	21.15 ± 2.18	0.978 a	10.15 ± 1.57	0.058 a	7.03 ± 2.22	0.292 a
Female	21.41 ± 2.18		9.82 ± 2.19		7.51 ± 1.20	
Age						
20–30 years	21.08 ± 2.21	0.076 b	9.86 ± 1.93	0.158 b	6.86 ± 2.20	0.009* b
31–40 years	21.54 ± 2.14		10.32 ± 1.56		7.75 ± 2.03	
41–50 years	2.167 ± 1.37		10.58 ± 1.38		7.74 ± 1.36	
Professional experience						
Less than 5 years	21.18 ± 2.20	0.923 b	9.91 ± 1.91	0.373 b	6.95 ± 2.23	0.055 b
5–10 years	21.40 ± 2.03		10.27 ± 1.52		7.56 ± 2.08	
11–15 years	21.28 ± 2.84		10.28 ± 1.81		7.78 ± 1.58	
More than 16 years	21.18 ± 1.94		10.54 ± 1.36		8.18 ± 1.17	
Education						
B. Pharm	22.60 ± 1.51	0.389 b	10.40 ± 1.67	0.142 b	7.40 ± 1.34	0.073 b
Pharm D	21.14 ± 2.26		9.87 ± 1.92		7.06 ± 2.14	
Higher degree	21.36 ± 2.01		10.44 ± 1.41		7.38 ± 2.17	
Other professional certificate/BCPS	22.00 ± 0.81		10.50 ± 1.29		9.75 ± 1.89	
Working setting						
Hospital Pharmacy	21.30 ± 2.02	0.014* b	10.07 ± 1.77	0.734 b	7.06 ± 1.20	< 0.001* b
Clinical Pharmacy	21.74 ± 2.30		10.13 ± 2.21		8.47 ± 2.18	
Community Pharmacy	19.75 ± 2.89		9.56 ± 1.63		6.93 ± 2.60	
Retail Pharmacy	20.81 ± 2.32		10.00 ± 1.48		6.31 ± 2.36	
Designation						
Trainee Pharmacist	20.87 ± 2.43	0.051 b	9.06 ± 2.46	0.012* b	6.42 ± 2.23	0.001* b
Resident Pharmacist	21.84 ± 1.74		10.48 ± 1.44		8.64 ± 1.68	
Staff Pharmacist	21.18 ± 2.06		10.00 ± 1.74		6.70 ± 2.02	
Assistant Manager Pharmacy	21.31 ± 2.70		10.43 ± 1.67		8.00 ± 2.75	
Manager Pharmacy	21.08 ± 2.04		10.52 ± 1.47		7.04 ± 1.79	
Chief Pharmacist	22.90 ± 1.66		11.00 ± 1.33		8.20 ± 2.82	
Director Pharmacy	19.44 ± 3.92		9.80 ± 1.09		7.20 ± 2.49	

a: Independent t test; b: One-way ANOVA; *p value < 0.05 statistically significant

Knowledge score: There was no significant difference in scores based on gender, age, professional experience, education level, or designation. However, there was a notable variation depending on the working setting. Pharmacists in clinical pharmacies scored higher (21.74 ± 2.30) than those in hospital pharmacies (21.30 ± 2.02), retail pharmacies (20.81 ± 2.32), and community pharmacies (19.75 ± 2.89) with a *p* value of 0.014.

Attitude score: The score was consistent across gender, age, professional experience, education level, and work setting. However, there was a significant difference based on designation. Chief pharmacists scored higher (11.00 ± 1.33) than their counterparts, with a *p* value of 0.012.

Perception score: The score remained steady concerning gender, professional experience, and education. However, there was a marked difference based on age, work setting, and designation. Pharmacists aged 31–40 scored the highest (7.75 ± 2.03) with a *p* value of 0.009. Those in clinical settings had a score of 8.47 ± 2.18, which was significantly higher than other settings (*p* value < 0.001). Finally, chief pharmacists led in scores with 8.20 ± 2.82, *p* value of 0.001.

The results demonstrate that while knowledge levels are satisfactory among the pharmacists, variations exist in their attitude and perceptions based on age, work setting, and designation.

**Fig. 1:**
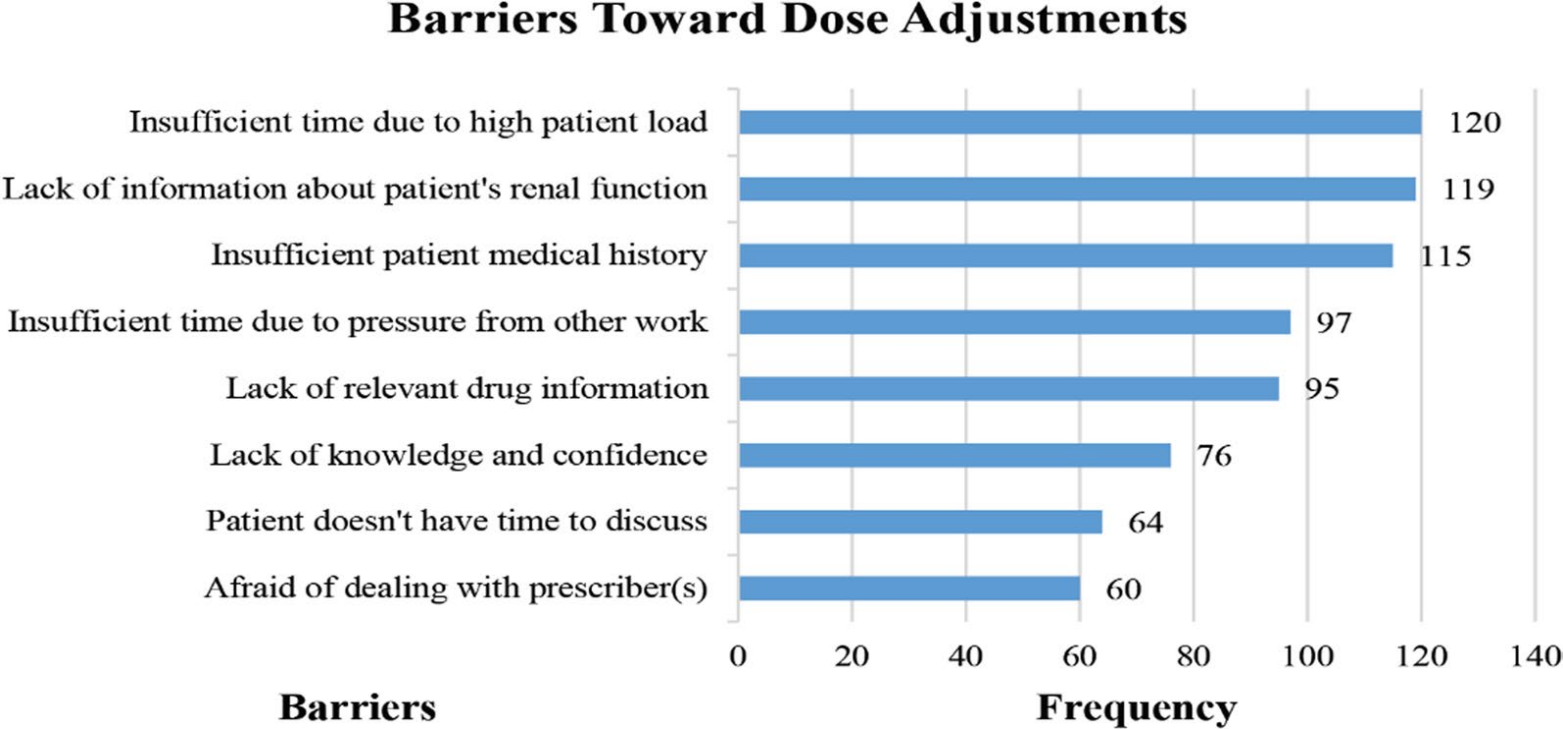
Significant carriers toward practicing of renal dose adjustment by pharmacists

The significant barriers toward practice of renal dose adjustment reported by pharmacist were insufficient time due to high patient load, lack of information about patient's renal function and insufficient patient medical history (as shown in Fig. 1). While majority of the pharmacist preferred (53%) online mode of training/Continuous medical education sessions followed by (57%) with face to face sessions.

Univariate analysis was performed to identify the potential factors independently associated with knowledge score, attitude score and perception score of pharmacists toward renal dosage adjustment, the potential factors were chosen on the basis of statistical significance having *p* value < 0.20 for multivariate analysis. The multivariate analysis revealed that there was a positive association between the perception score of pharmacists with gender; males have higher score as compared to females (β = 0.748; 95% CI 0.196; 1.300) (as shown in Table 6).

**Table 6:** Factors affecting knowledge score, attitude score and perception score of pharmacists toward renal dose adjustment on RDQ-13 scale

	Knowledge score				Attitude score				Perception score			
	Unadjusted		Adjusted		Unadjusted		Adjusted		Unadjusted		Adjusted	
	β	95% CI	β	95% CI	β	95% CI	β	95% CI	β	95% CI	β	95% CI
Gender	0.261	− 0.293; 0.815	–	–	− 0.333	− 0.792; 0.125	–	–	0.478	− 0.068; 1.023	0.748*	0.196; 1.300
Age	0.250	− 0.188; 0.688	–	–	0.417*	0.056; 0.777	0.482	− 0.209; 1.173	0.699*	0.274; 1.125	0.794	− 0.020; 1.607
Professional experience	0.061	− 0.277; 0.400	–	–	0.238	− 0.041; 0.517	− 0.155	− 0.682; 0.373	0.456*	0.126; 0.786	0.005	− 0.612; 0.623
Education	0.108	− 0.401; 0.617	–	–	0.418	− 0.001; 0.837	0.263	− 0.212; 0.738	0.462	− 0.038; 0.962	0.121	− 0.435; 0.677
Working setting	− 0.240	− 0.524; 0.038	0.240	− 0.524; 0.038	− 0.065	− 0.299; 0.169	–	–	− 0.074	− 0.353; 0.205		
Designation	0.033	− 0.174; 0.239	–	–	0.240	0.071; 0.409	–	–	0.110	− 0.094; 0.314		

Multivariate linear regression was used; Ref: Gender: Male = 0, female = 1; Age: 20–30 years = 0, 31–40 years = 1, 41–50 years = 2, 51–60 years = 3; Professional experience: < 5 years = 0, 5–10 years = 1, 11–15 years = 2, > 16 years = 3; Education: BSc Pharmacy/BPharm = 0, Pharm D = 1, Higher degree = 2, Other professional certificates/BCPS = 3; Working setting: Hospital pharmacy = 0, Clinical pharmacy = 1, Community pharmacy = 2, Retail pharmacy = 3; Designation: Trainee pharmacist = 0, Resident pharmacist = 1, Staff pharmacist = 2, Assistant manager pharmacy = 3, Manager pharmacy = 4, Chief pharmacist = 5, Director pharmacy = 6; *p < 0.05 statistically significant

## Discussion

Pakistan, classified as a low–middle-income country, is witnessing a sharp rise in chronic diseases, including diabetes mellitus, hypertension, and CKD. Factors, such as socio-economic status and a low literacy rate, coupled with a lack of adherence to preventive and management guidelines, render the population susceptible to these diseases. The latest statistics reveal that Pakistan has the highest diabetes rate at 30.8%, ranking it first above Kuwait, which has a rate of 24.9% [25]. The prevalence of diabetes mellitus surged from 1.7% to 17.1% between 2016 and 2019 [26]. This increasing trend is disconcerting. Similarly, the prevalence of hypertension in Pakistan is escalating rapidly. Notably, about 70% of patients remain unaware of their condition. Roughly 5.5 million males and 5.3 million females in Pakistan suffer from hypertension [27]. The escalating cases of diabetes and hypertension, potentially tied to urbanization [28], are significant contributors to CKD [29, 30]. With these underlying conditions on the rise, foreseeably, the necessity for renal dose adjustment will grow. It becomes imperative, therefore, to continually assess and enhance the proficiency of healthcare professionals, especially pharmacists, in ensuring correct dosing and rational treatment for CKD.

In our research, pharmacists working within a clinical pharmacy setup exhibited superior knowledge and perception scores related to renal dose adjustment compared to their peers in different settings. Given the scope of their roles, clinical pharmacists, especially those in inpatient settings, frequently engage in medication reviews and dose adjustments. These findings mirror the results from a study conducted in Malaysia [10]. Our data also revealed that chief pharmacists showcased a notably higher attitude score than other pharmacists. This could be attributed to their extensive professional experience and their influential role in guiding junior pharmacists. Their vast experience usually involves diverse training programs and the use of various decision support tools. This aligns with existing literature, suggesting that training, expert clinical support, and a robust clinical decision support system can mitigate drug-related issues in CKD patients [10, 31]. Moreover, the perception scores were significantly higher among resident pharmacists. One plausible explanation is that these pharmacists, often on temporary or contract-based positions, are keenly aware that their continued employment is performance-driven. This motivation might prompt them to meticulously adhere to standards and guidelines, resulting in elevated perception/practice scores. Owing to the limited research focusing on the knowledge, attitude, and perception of renal dose adjustment among pharmacists and healthcare professionals, drawing broad comparisons remains challenging. In our study, pharmacists aged between 41 and 50 years, possessing over 16 years of professional experience, displayed heightened knowledge, attitude, and perception scores compared to others. A study from Japan noted that pharmacists with ≥ 5 years of experience were 2.4 times more engaged in drug dose adjustments than those with ≤ 5 years of experience [17].

In our study, a majority of pharmacists indicated that among the various sources available for dosage adjustment, Medscape, the Renal Dosing Handbook, and mobile applications were their primary references. In contrast, other studies have reported that the Up-to-date and Micromedex databases were predominantly used by pharmacists for information regarding medication dosages and their adjustments [32, 33].

Our study also identified several barriers to the practice of renal dose adjustment. These included constraints on time due to a high patient load, the absence of information about a patient's renal function, and a lack of comprehensive patient medical history. Similar findings were echoed in a study that pointed out the challenges pharmacists face in procuring detailed patient medical histories, including renal function [10]. Another study reported barriers, such as difficulties in obtaining information on a patient's renal function, the oversight of prescriptions due to other pressing responsibilities, and a deficiency in the pharmacists' skills related to relevant pharmaceutical information [17]. The absence of a centralized national healthcare database or a formal renal registry in Pakistan complicates matters for healthcare professionals, including pharmacists, when addressing issues related to renal dose adjustment. Furthermore, having a sound understanding of CKD is paramount for healthcare professionals. An assessment conducted in Pakistan regarding pharmacists' knowledge of CKD revealed an adequate level of understanding [34]. In addition, the patients' self-perceived knowledge about CKD can influence the management of the disease. A study in Pakistan found that CKD patients' self-assessment of their understanding concerning the disease, medications, and lab investigations was suboptimal [35]. This underscores the urgency to bolster their knowledge for enhanced disease management and therapeutic outcomes. Given that CKD patients often grapple with multiple comorbidities leading to polypharmacy, they are at heightened risk for dosage errors and incorrect dose selections. Several studies have underscored the pivotal role pharmacists play in managing CKD and end-stage renal disease, enhancing patient outcomes, and refining care [14–16]. Collaborative efforts by clinical pharmacists, equipped with expertise in comprehensive drug management and therapeutics, have been recognized as crucial in advancing patient care [36, 37].

Considering the critical nature of renal dosage adjustment for patients with renal impairments, it is imperative that regular training sessions and continuous medical education/workshops be facilitated by the Ministry of Health. These sessions should target healthcare professionals, including pharmacists, to optimize the health and disease management of CKD patients. Our recommendations align with the findings from other studies, which suggest that training programs for healthcare professionals, including pharmacists, can significantly reduce the incidence of inappropriate dosage prescriptions [17, 32]. Moreover, introducing a computerized system that alerts pharmacists to renal impairments is essential for the accurate and timely implementation of drug dosage adjustments. However, implementing such a computerized alert system universally across all pharmacy setups might pose challenges [38]. As an alternative, an "alert card" system could be introduced. Under this system, CKD patients would be issued an alert card. This card would serve as a warning to healthcare professionals, ensuring pharmacists adjust medication dosages based on the individual patient's renal function test results.

### Strengths and limitations of the study

One significant merit of this study is its pioneering nature—it is the first to assess the knowledge, attitude, and perception toward renal dose adjustment among pharmacists in Pakistan. However, a potential limitation lies in its geographical scope. Given that the study was conducted solely in the Khyber Pakhtunkhwa province and Islamabad, its findings might not be representative of the entire pharmacist community in Pakistan.

## Conclusion

Pakistan, being at the forefront of this challenge, particularly with escalating rates of diabetes and hypertension, presents a unique context for this study. This pioneering research stands out as it marks the first comprehensive exploration into the knowledge, attitudes, and perceptions of renal dose adjustment among pharmacists in Pakistan. The findings from this study emphasize the gravity of ensuring accurate renal dosage adjustments for patients with compromised renal functions. These adjustments are paramount not only for achieving desired therapeutic outcomes but also for preventing adverse drug reactions and further progression of the disease. Based on the findings of this study, implementing targeted training programs to pharmacists, ensuring access to reliable resources, and promote research and dissemination of results in the field of renal pharmacotherapy are crucial steps toward improving population health outcomes. As Pakistan continues to witness an upsurge in chronic ailments, it is of paramount importance to intensify efforts toward optimizing renal pharmacotherapy.

## Data Availability

The data sets are available from the corresponding author upon reasonable request.
